# Adherence to the Provegetarian Food Patterns and Incidence of All-Cause Mortality in a Mediterranean Population: The SUN Cohort

**DOI:** 10.3390/nu17152472

**Published:** 2025-07-29

**Authors:** Ainara Martinez-Tabar, Miguel Ruiz-Canela, Vanessa Bullon-Vela, Carmen Sayon-Orea, Silvia Carlos, Miguel A. Martinez-Gonzalez, Maira Bes-Rastrollo

**Affiliations:** 1 Department of Preventive Medicine and Public Health, University of Navarra, 31008 Pamplona, Spain; amartinez.80@alumni.unav.es (A.M.-T.); mcanela@unav.es (M.R.-C.); mbullon@alumni.unav.es (V.B.-V.); msayon@unav.es (C.S.-O.); scarlos@unav.es (S.C.); mamartinez@unav.es (M.A.M.-G.); 2 Navarra Institute for Health Research (IdiSNA), 31008 Pamplona, Spain; 3 CIBER Physiopathology of Obesity and Nutrition (CIBEROBN), 28029 Madrid, Spain

**Keywords:** pro-vegetarian, dietary patterns, mortality, prospective, plant-based diets, plant-based index (PDI)

## Abstract

**Background and Objectives:** A provegetarian (PVG) food pattern, also known as a plant-based food pattern, which prioritizes the consumption of plant-based foods without completely excluding animal-based foods has been associated with health benefits. However, not all plant-based foods are healthy. We prospectively evaluated the association between different PVG food patterns and the risk of total mortality in the “Seguimiento Universidad de Navarra” (SUN) cohort. **Methods:** The SUN Project is a Mediterranean cohort study involving Spanish university graduates. A validated 136-item semi-quantitative food frequency questionnaire was used. A PVG food pattern, as previously proposed, was calculated assigning positive scores to plant-based foods and inverse scores to animal-based foods. Participants were categorized into quintiles based on their adherence to this pattern. Additionally, healthy and unhealthy PVG food patterns were derived. **Results:** Data from 17,989 participants with a mean baseline age (standard deviation) 38 (±12) years were analyzed. Over a mean follow-up period of 12 years, 460 deaths (2.6%) were recorded. Participants with higher adherence to the PVG food pattern (Q5) exhibited a 32% lower risk of total mortality [hazard ratio (HR): 0.68 (95% CI: (0.50–0.93); *p for trend* = 0.020] as compared to those with lower adherence (Q1), after adjusting for multiple confounders. This inverse association persisted for the healthy PVG food pattern [HR: 0.65 (95% CI: 0.47–0.90); *p for trend* = 0.016]. In contrast, the unhealthy PVG food pattern did not show any significant association with mortality [HR: 1.31 (95% CI: 0.94–1.83)]. **Conclusions:** Higher adherence to a PVG food pattern, which emphasizes the consumption of plant-based foods, reduces the risk of total mortality in the SUN cohort.

## 1. Introduction

Diets emphasizing plant-based foods over animal-based options, such as the Mediterranean diet, the healthy eating index 2015 (HEI-2015), or a plant-based diet, are crucial in preventing chronic diseases and mortality [1,2,3]. In recent years, there has been a notable increase in individuals adopting a plant-based diet due to concerns regarding animal welfare, the environment, and health [4,5]. Previous studies have demonstrated that a plant-based diet not only provides health benefits but is also environmentally sustainable [6,7]. Several types of plant-based diets vary in the degree of animal food restriction. A vegan diet entirely excludes all animal products; an ovo-lacto-vegetarian diet excludes all animal products except for eggs and dairy products; a pesco-vegetarian diet includes fish but excludes other meats, and a semi-vegetarian diet includes occasional meat consumption [8]. Some studies have assessed mortality among vegans, vegetarians, pesco-vegetarians, and semi-vegetarians, yielding contradictory results [9,10,11,12,13,14,15,16]. In the US-Adventist Health Study [10,16,17], vegetarians showed a lower risk of all-cause mortality compared to non-vegetarians. Conversely, other prospective cohort studies conducted in the US [11], Europe [12,13,15] and Australia [14] have not reported a significant association. Notably, some of these studies [9,10,15] involved health-conscious participants with healthier behaviors than those of the general population, which limits study comparability and the generalizability of findings to other populations. The nutritional quality of food patterns was not considered in these studies, and not all plant-based foods necessarily confer beneficial health effects [18]. Consumption of certain plant-based foods, such as vegetables, fruits, nuts, or whole grains, has been linked to reduced cardiovascular mortality risk [19,20], whereas intake of other plant-based foods, like sugar-sweetened beverages, has been associated with higher total mortality risk [21]. Therefore, it is crucial to assess the nutritional quality of plant-based foods and distinguish between healthy and unhealthy plant-based diets. Addressing this, Satija et al. developed in 2016 two plant dietary indices—the healthful plant-based diet index (hPDI) and the unhealthful plant-based diet index (uPDI)—which incorporate nutritional quality considerations [18]. In Spain, the prevalence of vegans and vegetarians remains low [4]. The highly restrictive nature of these diets and their difficulty to sustain long term pose challenges for many individuals in adhering to them. Therefore, a provegetarian (PVG) food pattern that emphasizes increasing plant-based foods while reducing but not entirely excluding animal-based foods, as Martínez-González et al. proposed [22], may be more feasible for the general population. Additionally, previous studies showed that high adherence to a plant-based diet that does not entirely eliminate animal-based foods is associated with reduced risk of all-cause mortality [23,24,25]. However, there is a lack of longitudinal studies with large sample size examining the association between pro-vegetarian food patterns and mortality in relatively young Mediterranean populations with repeated measurements of diet [26]. Therefore, to cover this scientific gap, this study aimed to assess the impact of three PVG food patterns (general, healthy, and unhealthy) on mortality using data from the “Seguimiento Universidad de Navarra” (SUN) Project.

## 2. Materials and Methods

### 2.1. Study Design and Population

The “Seguimiento Universidad de Navarra” (SUN) Project is a prospective, dynamic, and multipurpose cohort of Spanish university graduates. The recruitment began in December 1999, and it is still ongoing. The main objective is to study the impact of dietary and lifestyle habits on chronic diseases. The design, objectives, and methods have been described in detail elsewhere [27]. Participants complete a baseline questionnaire, and follow-up questionnaires are updated biennially, where the participants provide information on diet, lifestyle, and medical conditions.

From 1 December 1999 to 31 May 2022, 23,133 participants had completed the baseline questionnaire. For the present study, we excluded 234 subjects with insufficient follow-up time to complete the first follow-up questionnaire, 2123 subjects with total energy intake out of predefined limits (<500 or >3500 kcal/day for women, and <800 or >4000 kcal/day for men) [28] and 1188 subjects reporting a baseline medical diagnosis of cardiovascular disease (CVD), cancer or Type 2 diabetes mellitus (T2DM). Subjects with prevalent diseases were excluded because they may have changed their habits due to the diagnosis of these disorders, thereby originating a reverse causation bias. Additionally, 220 subjects with no answer for 70 or more items of the Food Frequency Questionnaire (FFQ) and 1379 subjects who were lost to follow-up were excluded (retention rate, 93%). For the present analyses, we used data from 17,989 participants of the SUN cohort (Figure S1).

Volunteers provided implied consent to take part in the study by completing and submitting the first self-administered questionnaire. All potential participants were informed of their right to decline participation or withdraw consent at any time without facing any consequences. To ensure the confidentiality and privacy of participants, the data were pseudonymized, with each individual assigned a study code number. This study was conducted in accordance with the principles of the Declaration of Helsinki. The voluntary completion of the baseline questionnaire was viewed as an indication of informed consent. The Research Ethics Committee at the University of Navarra approved this approach for obtaining informed consent from participants, and the Human Research Ethical Committee at the University of Navarra granted approval (2001/30).

### 2.2. Dietary Assessment

Dietary intake was measured at baseline and after 10 years of follow-up using a self-administered 136-item semiquantitative FFQ, which has been previously validated in Spain [29,30]. To assess the usual dietary intake (g/d) over the previous year, frequencies of intake were measured in 9 categories (ranging from “never or almost never” to “≥6 times a day”). Daily food consumption was calculated by multiplying the portion size by the consumption frequency for each food item.

An overall provegetarian (PVG) food pattern was calculated based on the methodology proposed by Martinez-Gonzalez et al. [22]. In addition, a healthy (hPVG) and unhealthy (uPVG) provegetarian food pattern was created according to the methodology followed by Oncina-Cánovas et al. [31] based on the plant-based diet scores from Satija et al. [18]. We created 19 food groups to calculate PVG, hPVG, and uPVG: vegetables, fruits, legumes, nuts, olive oil, whole grains, boiled or baked potatoes, French fries, coffee, refined grains, sugar sweetened beverages, fruit juices, pastries, dairy products, meat and meat products, fish or seafood, eggs, animal fats, and miscellaneous food. Of the 19 food groups, 8 were healthy plant-based food groups (vegetables, fruits, legumes, nuts, olive oil, whole grains, boiled or baked potatoes, and coffee), 5 were unhealthy plant-based food groups (refined grains, French fries, sugar-sweetened beverages, fruit juices, and pastries), and 6 were animal-based food groups (dairy products, meat or meat products, fish or seafood, eggs, animal fats, and miscellaneous food). To create each provegetarian food pattern, the intake of each food group (g/d) was adjusted for total energy intake using the residual method separately for men and women [28]. The energy-adjusted estimates (residuals) were categorized into quintiles, and each quintile was assigned a score ranging from 1 to 5. To build the PVG food pattern, plant-based food groups were scored positively, while animal-based food groups were scored reversely. In the case of hPVG, healthy plant-based food groups received positive scores, whereas unhealthy plant-based food groups and animal food groups received reverse scores. Finally, for the uPVG food pattern, unhealthy plant-based food groups were given positive scores, and healthy plant-based food groups and animal food groups were given reverse scores. The quintile values of the food groups were summed to obtain the overall, healthy, and unhealthy PVG food pattern. Their final score could range from 19 (lowest adherence) to 95 (highest adherence). Table S1 describes the food items included in each food pattern (PVG, hPVG and uPVG) and their scoring criteria. Participants were divided into quintiles according to their adherence to each PVG food pattern. Neither the consumption of margarine nor the consumption of alcoholic beverages was included in the score. The composition of margarine has changed over time (from being high in trans-fatty acid to being high in unsaturated fatty acid) [18]. The consumption of alcoholic beverages and its effect on health is still controversial, so it was also not included in the score. However, alcohol consumption (g/d) was included as an adjustment variable in the main analyses, and margarine was included as an additional covariate in sensitivity analysis.

### 2.3. Outcome Assessment

We identified all deaths occurring between the baseline questionnaire reception and May 2022. To be able to contact all the participants at any time during the follow-up period, we contacted them on several occasions on a yearly basis, to update three alternative postal addresses. When the postal contact failed, we used their telephone number and email addresses to contact them. Additionally, to obtain information from participants who did not complete the follow-up questionnaires, information was periodically exchanged with the alumni associations of the University of Navarra and other professional associations. Most of the deaths identified (>85%) in the SUN cohort were reported by participants’ relatives, professional associations, or ordinary mail. Also, The National Death index was consulted every year to confirm the vital status of the participants and to complete the information on mortality, including the cause of death, classified according to the 10th Revision of the International Classification of Diseases (ICD-10).

### 2.4. Assessment of Other Variables

The baseline questionnaire also collected information about sociodemographic characteristics (sex, age, marital status, and education level), health-related habits (alcohol intake, smoking status, and physical activity), anthropometric measurements (weight and height), dietary factors (snacking between meals and following a special diet) and clinical variables (prevalence of cancer, CVD, T2DM, hypertension, and hypercholesterolemia). We calculated BMI using self-reported weight (kg) divided by the square of self-reported height (m). Anthropometric variables were previously validated in the SUN cohort [32].

### 2.5. Statistical Analysis

Participants were categorized into quintiles based on their adherence to each food pattern.

We used Cox regression models to test the association between each PVG food pattern and all-cause mortality. Hazards ratios (HR) and 95% confidence intervals (CI) were calculated using the lowest quintile of adherence to each dietary pattern as the reference category with age as underlying time-variable. Exit time was defined as date of death or date when the last follow-up questionnaire was completed.

The Cox regression models were adjusted for several potential confounders. Model 1 was adjusted for age (as underlying variable), sex, BMI (kg/m^2^, linear and quadratic terms, continuous), marital status (married, yes/no) educational level (graduate, postgraduate, and doctorate), physical activity (METs-h/week, continuous), total energy intake (five categories), total cumulative exposure to cigarette smoking (four categories), smoking status (never, current, and former smoker), following a special diet (yes/no) and snacking between meals (yes/no). Model 2 was also adjusted for prevalent hypertension (yes/no) and prevalent hypercholesterolemia (yes/no). Analyses were stratified by recruitment period (5-year period) and by age group (10-year period). Additionally, tests of linear trend across successive quintiles of adherence were performed assigning the median value to each quintile category and treating the variable as continuous in the respective Cox regression model. Multivariate Cox regression models were performed using the cumulative dietary averages from baseline and 10-year follow-up information.

To assess the degree of overlap between each PVG food pattern and the Mediterranean diet, we calculated Pearson’s correlation coefficients between each PVG food pattern and MedDiet 9-point score [33]. The percentage of participants who were in the same quintile for both food patterns was also calculated.

Although we adjusted for a wide range of potential confounders, we cannot rule out residual confounding. PVG food patterns may be associated with other dietary or lifestyle factors. To address this issue, we estimated the E value suggested by VanderWeele [34]. The E-value represents the minimum strength of association, on the risk ratio scale, that an unmeasured confounder would need to have with both the exposure and outcome to fully explain away a specific exposure–outcome association, conditional on the measured covariates.

We assessed the interaction between quintiles of adherence to each food pattern and sociodemographic characteristics or health-related habits using a likelihood ratio test (four degrees of freedom) that compared the multivariable Cox regression model (model 2) and the same model with interaction product-terms. Possible effect modification of each pattern by sex, age (≥55 years and <55 years at recruitment), BMI (<25 and ≥25 kg/m^2^) physical activity (above median/below median), and smoking status (ever smoker or never smoker) was tested.

We conducted sensitivity analyses by rerunning all the models under different assumptions to evaluate the robustness of our findings: excluding participants with energy intake <5th and >95th percentiles; excluding deaths in the first 2 years of follow-up (to limit the reverse causation); without excluding participants with cardiovascular disease, diabetes or cancer; excluding adjustment for special diets; including only cancer deaths or CVD deaths. We also adjusted the results for margarine intake, vegetable fat and oil intake, or prevalent depression.

Analyses were conducted using STATA/SE version 15.0 (StataCorp, College Station, TX, USA) with a statistical significance of 5% based on two-tailed tests.

## 3. Results

We analyzed data from 17,989 (10,961 women and 7028 men) participants. The mean age and standard deviation at baseline were 38 ± 12 years. After a median follow-up of 12 years (234,867 person–years of follow-up), we identified 460 deaths including 227 deaths due to cancer (49%), 147 (32%) due to other causes, and 86 due to CVD (19%).

The baseline characteristics of participants according to quintiles of each PVG food pattern (PVG, hPVG and uPVG) are summarized in Table 1. Participants across quintiles of hPVG food pattern were older, more likely to be married, reported a higher physical activity, were more likely to follow a special diet, had a greater adherence to the Mediterranean diet, and consumed more vitamin and mineral supplements. They were less likely to be current smokers, to spend more hours watching television, to consume snacks between meals or alcohol. Inversely, subjects in the highest quintile of the uPVG food pattern were more likely to be younger, to smoke, to watch television, to snack, and to consume alcohol. Also, they were less likely to be married, to practice physical activity, to follow a special diet, to have a high adherence to the Mediterranean diet, and to consume supplements.

Baseline dietary information from the SUN Project participants according to quintiles of each PVG food pattern (PVG, hPVG and uPVG) is described in Table S2. A higher intake of carbohydrate and fiber and a lower intake of protein, fat, and fatty acids (saturated, monounsaturated, polyunsaturated, and trans fatty acid) was observed in the subjects with the highest adherence to the hPVG pattern. Subjects with higher adherence to the uPVG pattern reported a higher intake of carbohydrates, saturated fatty acid, polyunsaturated fatty acid, monounsaturated fatty acid, trans fatty acid, and a lower intake of proteins and fibers. Participants in the highest category of the uPVG food pattern reported the highest consumption of refined grains, French fries, sugar-sweetened beverages, fruit juices, and pastries, as expected.

**Figure 1:**
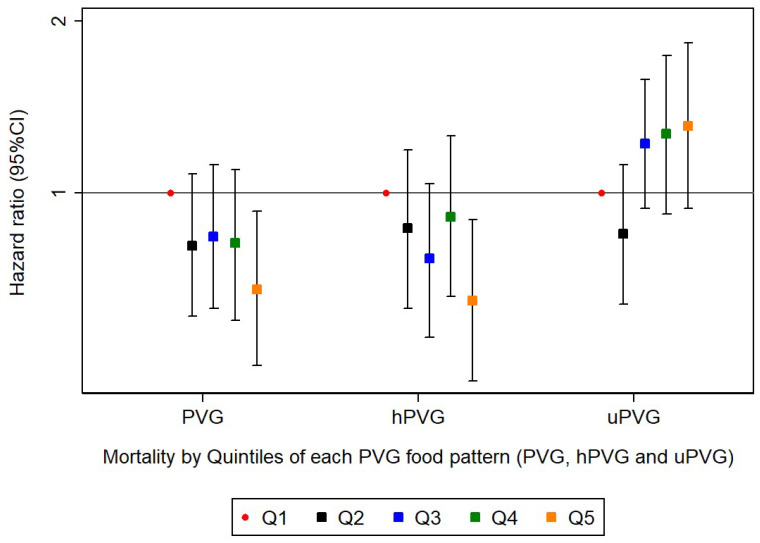
Risk of all-cause mortality (adjusted* HR and 95% CI) according to quintiles of each provegetarian food pattern. *Adjusted for age, sex, BMI (kg/m^2^, linear and quadratic terms, continuous), marital status (dichotomous) years of university education (three categories), physical activity (continuous), total energy intake (five categories), cumulative smoking habit (packs–years, four categories), smoking status (three categories), alcohol intake (g/d), following a special diet (dichotomous), snacking (dichotomous), prevalent hypertension (dichotomous) and prevalent hypercholesterolemia (dichotomous). Abbreviations: PVG: provegetarian; hPVG: healthful provegetarian; uPVG: unhealthful provegetarian.

Table 2 and Figure 1 presents the associations between each provegetarian food pattern (PVG, hPVG, and uPVG) and all-cause mortality in the SUN cohort study. Compared to subjects in the lowest quintile, those in the highest quintile of the PVG food pattern had 32% lower total mortality after multivariable adjustment (model 2) [HR: 0.68 (95% CI: 0.50–0.93)]. The multivariable model (model 2) showed a significant dose–response relation between PVG food pattern and all-cause mortality (*p for trend* = 0.020). Similar results were observed for the association between the hPVG food pattern and all-cause mortality [hPVG: HR _Q5 vs. Q1_: 0.65 (95% CI: 0.47–0.90)]. The hPVG food pattern was inversely associated with all-cause mortality in the model adjusted for sociodemographic factors, lifestyle risk factors, and medical conditions at baseline (model 2) [HR: 0.65 (95% CI: (0.47–0.90); *p for trend* = 0.016]. No significant association was found between uPVG food pattern and total mortality [HR: 1.31 (95% IC: 0.94–1.83)], although it presented a higher risk dose–response relationship (*p* linear trend = 0.037). Cox proportional hazard models were fitted with repeated measurements using cumulative average dietary information after 10 years of follow-up.

Associations of PVG and hPVG food patterns with mortality remained consistent and significant [PVG: HR _Q5 vs. Q1_: 0.69 (95% CI: 0.51–0.93)]; [hPVG: HR _Q5 vs. Q1_: 0.66 (95% CI: 0.48–0.91)], suggesting that these associations are robust. Moreover, the uPVG was associated with a borderline statistically significantly increased risk of all-cause mortality [uPVG: HR _Q5 vs. Q1_: 1.40 (95% CI: 1.00–1.97)], with a significant dose–response relationship (*p* for trend = 0.019) when using cumulative average dietary information.

**Figure 2:**
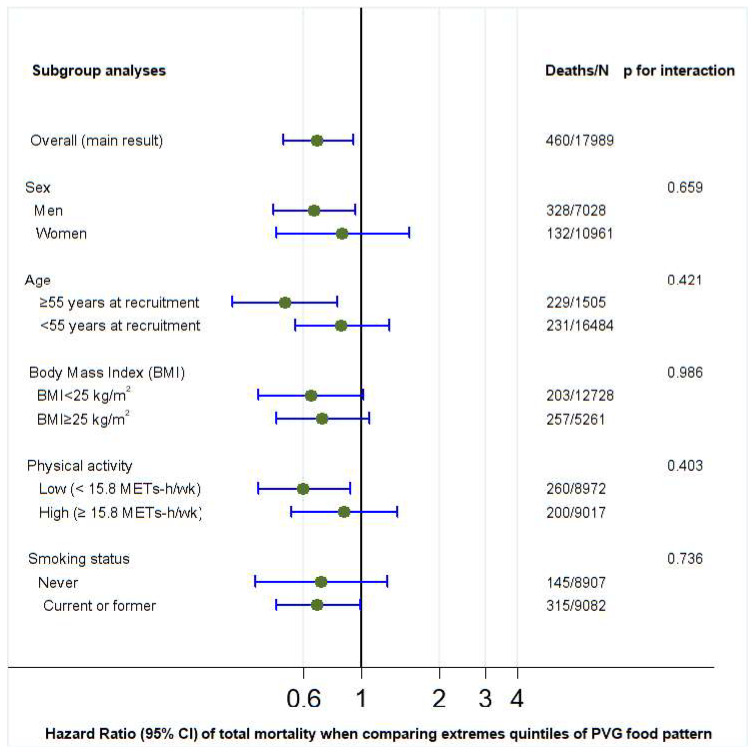
Subgroup adjusted* analyses of the association between provegetarian food pattern and all-cause mortality in different scenarios (highest versus lowest quintile). *Adjusted for age, sex, BMI (kg/m^2^, linear and quadratic terms, continuous), marital status (dichotomous), years of university education (three categories), physical activity (continuous), total energy intake (five categories), cumulative smoking habit (packs–years, four categories), smoking status (three categories), alcohol intake (g/d), following a special diet (dichotomous), snacking (dichotomous), prevalent hypertension (dichotomous), and prevalent hypercholesterolemia (dichotomous). Abbreviations: PVG: provegetarian.

**Figure 3:**
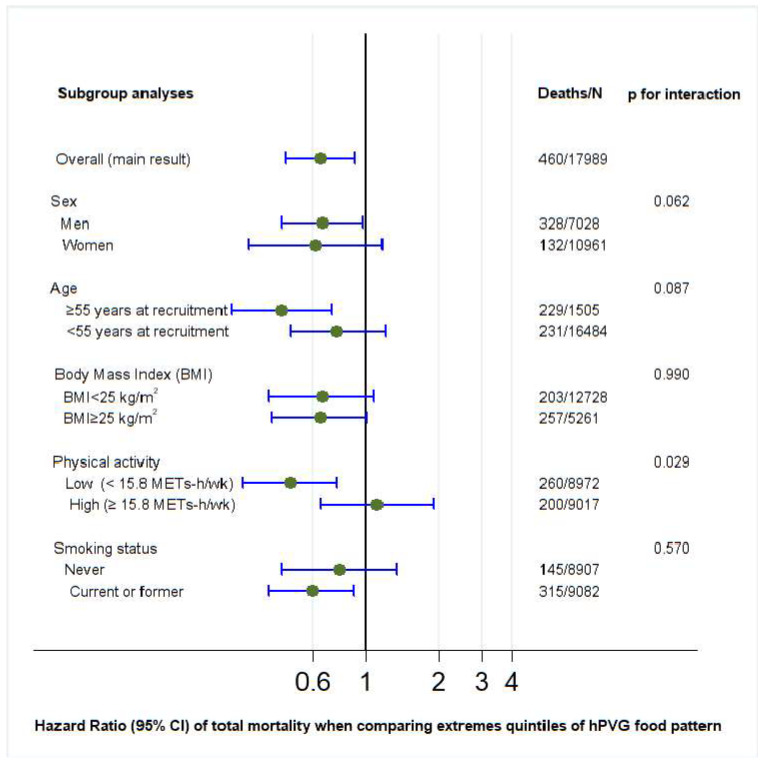
Subgroup adjusted* analyses of the association between healthful provegetarian food pattern and all-cause mortality in different scenarios (highest versus lowest quintile). *Adjusted for age, sex, BMI (kg/m^2^, linear and quadratic terms, continuous), marital status (dichotomous), years of university education (three categories), physical activity (continuous), total energy intake (five categories), cumulative smoking habit (packs–years, four categories), smoking status (three categories), alcohol intake (g/d), following a special diet (dichotomous), snacking (dichotomous), prevalent hypertension (dichotomous), and prevalent hypercholesterolemia (dichotomous). Abbreviations: hPVG: healthful provegetarian.

**Figure 4:**
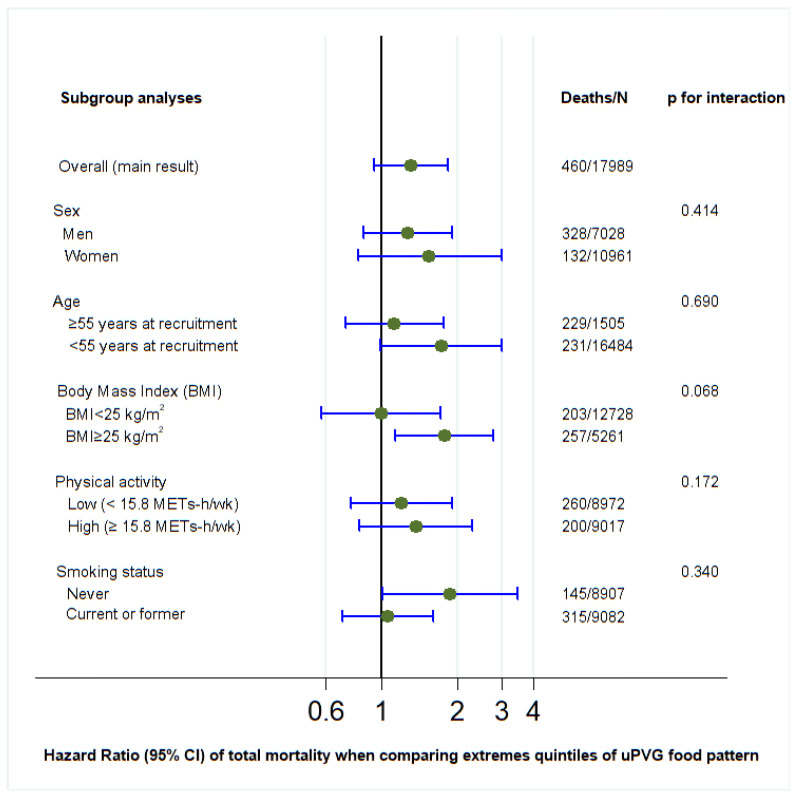
Subgroup adjusted* analyses of the association between unhealthful provegetarian food pattern and all-cause mortality in different scenarios (highest versus lowest quintile). *Adjusted for age, sex, BMI (kg/m^2^, linear and quadratic terms, continuous), marital status (dichotomous), years of university education (three categories), physical activity (continuous), total energy intake (five categories), cumulative smoking habit (packs-years, four categories), smoking status (three categories), alcohol intake (g/d), following a special diet (dichotomous), snacking (dichotomous), prevalent hypertension (dichotomous) and prevalent hypercholesterolemia (dichotomous). Abbreviations: uPVG: unhealthful provegetarian.

*p* values for interaction were calculated for PVG, hPVG, and uPVG with sociodemographic and lifestyle variables (sex, age, BMI, physical activity, and smoking status) using the likelihood ratio test (Figure 2, Figure 3 and Figure 4). Regarding the hPVG pattern, the interaction with physical activity was significant (*p* < 0.029). The HR for those who practice exercise below the median (15.8 METS) was 0.49 (95% CI: 0.31–0.76) and 1.11 (95% CI: 0.65–1.90) for those above the median. The rest of the interactions were not significant (*p* > 0.05). We did not find any interaction for PVG and uPVG food patterns with sociodemographic and lifestyle variables (Figure 2 and Figure 4).

Sensitivity analyses were performed by repeating the fully adjusted model in different scenarios comparing the highest quintile with the lowest quintile of each food pattern to assess the robustness of our results (Table 3). Similar associations of PVG and hPVG food patterns with total mortality were found after using 5th and 95th centiles as limits for total energy intake, excluding deaths in the first two years, excluding subjects following a special diet and after adjusting for margarine intake, other vegetable fat and oil intake, or prevalent depression. However, no association was found when including only deaths from cancer or CVD.

Additionally, the E value proposed by VanderWeele et al. was calculated. For the multivariate adjusted results in our study, the E-value was 2.302 for the estimate and 1.360 for the CI for the PVG, while for the hPVG, the E-value was 2.449 for the estimate and 1.462 for the CI. The observed HR of 0.68 in our analysis may be attributed to an unmeasured confounder associated with PVG food pattern and mortality, both having a HR of 2.302, in addition to the measured confounders. However, a less influential confounder would be insufficient to account for this finding. Similarly, the lowest CI could be adjusted to include the null due to an unmeasured confounder linked to both the PVG food pattern and mortality, each with a HR of 1.360, in addition to the measured confounders. Nevertheless, a weaker confounder would also not suffice.

Table S3 shows the Pearson correlation coefficients between each PVG food pattern and the Mediterranean diet. No major degree of overlap was found between the Mediterranean food pattern and the PVG or hPVG food pattern. The Pearson’s correlation coefficients between these dietary patterns were 0.38 and 0.54, respectively. Additionally, 29.3% of the participants were classified in the same quintile of the Mediterranean and PVG food pattern, while 33.8% of the individuals were classified in the same quintile of the Mediterranean and hPVG food pattern.

## 4. Discussion

In this prospective cohort study formed of relatively young Spanish university graduates, greater adherence to the PVG and hPVG food patterns was significantly associated with a decrease in all-cause mortality with a linear dose–response relationship, even after adjusting for potential confounding factors. These associations remained consistent across sensitivity analyses and repeated measurements. A borderline statistically significantly direct relationship was observed between the uPVG food pattern and total mortality in repeated measurements.

In our Mediterranean cohort, participants with higher adherence to the PVG food pattern showed a 32% lower risk of all-cause mortality. Our results are generally consistent with previous studies that evaluated this association [24,26,35,36,37,38,39,40,41]. In the Prevención con Dieta Mediterranea (PREDIMED) study, among an older Mediterranean population at high cardiometabolic risk, Martínez-González et al. showed that greater adherence to the PVG food pattern was associated with a 41% reduction in all-cause mortality [22]. On the other hand, we observed that the highest quintile of hPVG food pattern was associated with a 35% lower risk of total mortality. Our findings are in line with the study conducted by Delgado et al. the ENRICA study, a Spanish based population cohort. An increase of 10-point in hPVG food pattern was associated with a 14% lower risk of all-cause mortality, although the sample size was smaller than in our study [42]. Moreover, in a cross-sectional study conducted by Oncina-Cánovas et al. using data from the European Eye Study (EUREYE) in Spain with 597 older participants, those in the second tertile of adherence to the hPVG food pattern exhibited a significantly lower risk of total mortality compared to those in the lowest tertile [43]. These results are in line with other studies conducted in non-Mediterranean populations [24,36,37,39,40,44,45,46,47,48].

Some studies examining the effect of PVG food patterns on mortality found that greater adherence to the uPVG food pattern was associated with an increased risk of mortality [26,40,41,43,46,49], whereas others did not find this relationship [36,42,45,50]. In our analyses, when we used the cumulative average of dietary intake, uPVG was associated with a borderline statistically significantly increased risk of all-cause mortality. However, most previous studies [23,24,25,42,43,44,45,46,47,50] only assessed dietary intake at baseline, which limits comparability with these results. Some studies with repeated assessment of dietary information showed that the uPVG food pattern was associated with increased risk of all-cause mortality [35,37], while others did not observe an association [36] or did not evaluate the uPVG food pattern [2]. As dietary habits change over time, more studies are needed to evaluate how these changes relate to mortality [51].

Several mechanisms may explain the observed relationship between the adherence to the hPVG food pattern and lower risk of all-cause mortality. Previous studies showed that a healthy plant-based diet has a beneficial effect on cardiovascular disease and its risk factors [18,31,52,53,54]. These diets are typically rich in fiber, polyphenols, and unsaturated fatty acids—bioactive components known to reduce oxidative stress and inflammation [55,56,57]. Such compounds are abundant in key plant-based food groups: fruits and vegetables provide a wide range of polyphenols; whole grains supply substantial amounts of dietary fiber and resistant starch; and legumes and nuts are major sources of plant-based proteins and unsaturated fats. Together, these nutrients and compounds likely contribute to the observed protective effects on mortality [58,59,60,61,62]. In the NHANES study, Brigitte Wang et al. demonstrated that a healthy plant-based diet was linked to lower systemic inflammation [63]. In the same study, Hao-Wei et al. suggested that C-reactive protein and γ-glutamine transaminase might mediate the relationship between a plant-based diet and all-cause mortality in individuals with diabetes [46]. Another mechanism that could explain this relationship is the effect of plant-based foods on gut microbiota and their metabolites [64,65].

The Mediterranean diet, which prioritizes a higher intake of plant-based foods, has also been inversely associated with mortality risk [66,67]. Both the Mediterranean diet and healthy PVG food pattern are characterized by high intakes of fruits, vegetables, legumes, and nuts and low consumption of red meat, processed meat, butter, sugar sweetened beverages, and commercial bakery such as biscuits and pastries. However, the Mediterranean diet considers the quality of animal-based foods, while the PVG food pattern does not. Our findings showed that in the SUN cohort, there was not a large degree of overlap between the Mediterranean diet and the PVG food pattern. These findings may be due to the fact that the PVG pattern negatively scores fish consumption, in contrast to the Mediterranean diet, which assigns a positive score to moderate wine consumption and does not consider coffee or potatoes.

Our results are aligned with the current recommendations of the EAT-Lancet initiative, which advocates for high consumption of vegetables, fruits, whole grains, legumes, nuts, and unsaturated oils; moderate consumption of seafood and poultry; and low or no consumption of red meat, processed meat, added sugar, refined cereals, and starchy vegetables for health and environmental benefits [68,69]. Similar results emerged from studies evaluating the impact of planetary health diet scores, derived from the EAT-Lancet dietary report, on mortality [70,71].

Certain limitations of our study should be acknowledged. First, we used information from a self-reported semi-quantitative FFQ which may introduce some non-differential measurement error. Nevertheless, the FFQ has been repeatedly validated in Spain with good reproducibility and relative validity [27,29,30]. We also gathered self-reported information about other measurements such as BMI that have been previously validated [32]. Moreover, the high motivation and educational level of our participants improve the quality of the self-reported data. Second, we cannot rule out the possibility of residual confounding. Nevertheless, we adjusted for multiple potential confounders in various multivariable models, and the findings remained robust. Additionally, the E values for the point estimate supported the association between PVG food patterns and all-cause mortality. The point estimate could only be theoretically explained by an unmeasured confounder with a HR of at least 2.302-fold for all-cause mortality and PVG food pattern. Third, the SUN cohort is not representative of the general population. Participants in our cohort were relatively young and with low prevalence of chronic diseases, which compromises the external validity of our results, to generalize the findings to an elderly population with a high prevalence of chronic diseases. However, the restriction to highly educated participants reduces the possible confounding related to educational level [72], improving the internal validity of our findings. Moreover, the generalizability of the results should be based on biological plausibility and not only on statistical representativeness. Fourth, as the cohort comprises relatively young participants with a low prevalence of chronic diseases, the total number of deaths observed was low. This limited number of events may compromise the statistical power of the study, particularly affecting subgroup analyses. Therefore, the results of these analyses should be interpreted with caution. Future research with larger numbers of events is warranted to confirm and extend our findings. Fifth, we should acknowledge that not all animal-based foods are equal in nutritional quality; however, we did not distinguish the quality of animal-based foods because the aim of the provegetarian score is to capture adherence to plant-based diets as a whole with preference for plant-based food. Nonetheless, we acknowledge that such scoring may misrepresent certain balanced, health-promoting dietary patterns. However, we followed this methodology for the sake of consistency with the previous literature and to enable comparability across studies. Sixth, voluntary participation may have introduced self-selection bias, as individuals with greater health awareness or healthier lifestyles are more likely to enroll. This inherent limitation of cohort studies based on voluntary recruitment should be taken into account.

Although we did not collect direct measures of socioeconomic status (SES) such as income, since all participants are university graduates, we used the restriction method to adjust for SES taking education as a surrogate of SES [73]. We are confident that SES cannot be a major confounder. In addition, to avoid residual confounding we adjusted the analyses for participants’ years of education.

The major strengths include a large sample size, prospective design (which limits the possibility of reverse causality), long follow-up with repeated measures of diet although only at 10 years, good retention rate (93%), adjustment for numerous confounding factors, the verification of mortality cases by medical records or consultation of the National Death Index and the validation of some measurements. Another strength is the inclusion of a wide range of sensitivity analyses to test the robustness of our results. Moreover, repeated measurements were performed to assess how changes in dietary habits are associated with mortality. Lastly, although numerous studies have evaluated the association between the provegetarian food patterns and mortality, this is the first large prospective cohort study conducted in a relatively young Mediterranean population with repeated measurements of diet.

## 5. Conclusions

Better adherence to the PVG and hPVG food patterns was associated with a reduced risk of all-cause mortality in the SUN cohort. A borderline association was observed between the uPVG food pattern and mortality in this relatively young Mediterranean population. Further research in similar populations is needed to confirm and explain these associations.

## Figures and Tables

**Table 1:** Baseline characteristics of participants according to quintiles (Q) of the provegetarian (PVG), healthful provegetarian (hPVG), and unhealthful provegetarian (uPVG) food patterns in the SUN Project.

	PVG			hPVG			uPVG		
Variables	Q1	Q2–Q4	Q5	Q1	Q2–Q4	Q5	Q1	Q2–Q4	Q5
*n*	4278	10,215	3496	4090	10,634	3265	4130	10,559	3300
Provegetarian score range	29–52	53–62	63–81	34–51	52–63	64–86	31–51	52–63	64–87
Age (years)	35.3 (11.4)	37.5 (11.7)	39.7 (11.8)	32.9 (9.8)	37.6 (11.6)	42.5 (12.2)	41.0 (12.3)	37.2 (11.6)	33.8 (10.2)
Female (%)	59.9	61.8	59.7	60.8	61.2	60.2	60.9	61.1	60.4
Married (%)	44.3	50.2	53.2	37.6	51.03	58.5	57.1	49.0	40.8
Years of university education	5.0 (1.4)	5.0 (1.5)	5.2 (1.6)	5.0 (1.5)	5.1 (1.5)	5.1 (1.5)	5.1 (1.5)	5.1 (1.5)	5.0 (1.4)
BMI (kg/m^2^)	23.3 (3.4)	23.5 (3.5)	23.5 (3.4)	23.1 (3.4)	23.5 (3.5)	23.6 (3.5)	23.9 (3.5)	23.4 (3.5)	23.0 (3.3)
Physical activity (METs-h/week)	21.3 (23.4)	21.6 (22.3)	23.1 (24.5)	19.9 (22.2)	21.5 (22.3)	25.6 (25.7)	23.7 (23.7)	21.7 (23.1)	19.9 (21.5)
Cumulative smoking habit (pack-years)	5.3 (9.4)	5.8 (9.4)	6.6 (9.7)	4.0 (7.5)	6.0 (9.7)	7.5 (10.5)	7.3 (10.5)	5.7 (9.3)	4.4 (8.3)
Smoking (%)									
Never smoker	52.1	49.6	46.0	56.3	48.5	44.2	44.7	49.7	55.1
Current smoker	23.2	22.0	21.3	23.5	22.9	18.1	20.3	22.5	23.4
Former smoker	24.6	21.3	32.7	20.2	28.6	37.7	35.0	27.8	21.6
Television watching (h/d)	1.7 (1.2)	1.6 (1.1)	1.5 (1.1)	1.7 (1.3)	1.6 (1.1)	1.5 (1.1)	1.6 (1.1)	1.6 (1.1)	1.7 (1.2)
Snacking between meals (%)	37.8	33.1	29.3	41.4	32.5	26.8	26.1	33.3	43.4
Following a special diet (%)	5.6	7.6	10.0	4.6	7.4	12.0	12.3	7.2	3.2
Alcohol intake (g/d)	7.0 (11.2)	6.5 (9.7)	6.2 (8.3)	5.5 (7.9)	6.6 (9.9)	7.7 (11.5)	6.2 (8.8)	6.6 (9.5)	6.9 (11.9)
Adherence to Mediterranean diet, Trichopoulou (%)									
Low (0–3)	56.9	35.2	17.1	66.8	34.2	7.7	20.1	38.4	52.9
Medium (4–5)	34.3	40.9	38.8	28.1	44.1	35.3	39.9	39.8	34.9
High (6–9)	8.9	24.0	44.1	5.1	21.6	57.0	40.1	21.9	12.2
Supplement use (%)	17.9	18.5	19.7	17.7	18.3	20.8	19.2	18.3	18.7
Hypertension at baseline (%)	8.4	9.7	10.4	6.7	9.8	12.2	12.1	9.2	7.4
Hypercholesterolemia at baseline (%)	12.5	16.3	21.0	11.4	16.3	22.4	19.6	16.1	12.9

Categorical variables are described as percentages and quantitative variables as means (standard deviations). Abbreviations: PVG: provegetarian; hPVG: healthful provegetarian; uPVG: unhealthful provegetarian; BMI: body mass index.

**Table 2:** Cox proportional HRs and 95% CIs for all-cause mortality according to quintiles of each provegetarian food pattern.

**PVG Food Pattern**	**Q1**	**Q2**	**Q3**	**Q4**	**Q5**	***p for trend***
*n*	4278	3957	3365	2893	3496	
Deaths/person-years	105/57,223	103/52,188	95/43,815	76/37,463	81/44,176	
Sex and age adjusted	1.00 (ref.)	0.85 (0.65–1.12)	0.88 (0.67–1.16)	0.84 (0.63–1.13)	0.71 (0.53–0.95)	0.030
Model 1	1.00 (ref.)	0.81 (0.61–1.07)	0.84 (0.63–1.12)	0.81 (0.60–1.10)	0.69 (0.51–0.93)	0.023
Model 2	1.00 (ref.)	0.81 (0.61–1.08)	0.84 (0.63–1.12)	0.82 (0.60–1.10)	0.68 (0.50–0.93)	0.020
Repeated measurements of diet	1.00 (ref.)	0.79 (0.59–1.05)	0.82 (0.61–1.09)	0.79 (0.59–1.06)	0.69 (0.51–0.93)	0.034
**hPVG Food Pattern**	**Q1**	**Q2**	**Q3**	**Q4**	**Q5**	***p for trend***
*n*	4090	3725	3889	3020	3265	
Deaths/person-years	72/54,737	92/48,522	98/51,384	96/38,828	102/41,394	
Sex and age adjusted	1.00 (ref.)	0.97 (0.71–1.32)	0.84 (0.62–1.14)	0.95 (0.69–1.31)	0.71 (0.52–0.98)	0.030
Model 1	1.00 (ref.)	0.88 (0.64–1.20)	0.78 (0.57–1.05)	0.92 (0.67–1.26)	0.67 (0.49–0.92)	0.022
Model 2	1.00 (ref.)	0.87 (0.63–1.19)	0.77 (0.56–1.04)	0.91 (0.66–1.26)	0.65 (0.47–0.90)	0.016
Repeated measurements of diet	1.00 (ref.)	0.78 (0.57–1.08)	0.75 (0.54–1.02)	0.86 (0.63–1.18)	0.66 (0.48–0.91)	0.059
**uPVG Food Pattern**	**Q1**	**Q2**	**Q3**	**Q4**	**Q5**	***p for trend***
*n*	4130	3740	3779	3040	3300	
Deaths/person-years	138/52,033	86/48,611	103/49,829	69/40,134	64/44,259	
Sex and age adjusted	1.00 (ref.)	0.82 (0.62–1.09)	1.18 (0.91–1.52)	1.17 (0.87–1.59)	1.22 (0.90–1.67)	0.068
Model 1	1.00 (ref.)	0.84 (0.64–1.12)	1.23 (0.94–1.59)	1.27 (0.93–1.74)	1.29 (0.92–1.81)	0.041
Model 2	1.00 (ref.)	0.85 (0.64–1.12)	1.22 (0.94–1.58)	1.27 (0.92–1.74)	1.31 (0.94–1.83)	0.037
Repeated measurements of diet	1.00 (ref.)	1.06 (0.81–1.39)	1.15 (0.88–1.50)	1.37 (1.00–1.87)	1.40 (1.00–1.97)	0.019

All the models were stratified by age groups (10-year periods) and recruitment period (5-year periods). Model 1: adjusted for age, sex, BMI (kg/m^2^, linear and quadratic terms, continuous), marital status (dichotomous) years of university education (five categories), physical activity (continuous), total energy intake (five categories), cumulative smoking habit (packs–years, four categories), smoking status (three categories), alcohol intake (g/d), following a special diet (dichotomous), and snacking (dichotomous). Model 2: additionally, adjusted for prevalent hypertension (dichotomous) and prevalent hypercholesterolemia (dichotomous).

**Table 3:** Sensitivity analyses for the association between each provegetarian food pattern and all-cause mortality in different scenarios (highest versus lowest quintile).

	*n*	Deaths	PVG	hPVG	uPVG
			HR (95% CI)	HR (95% CI)	HR (95% CI)
Overall	17,989	460	0.68 (0.50–0.93)	0.65 (0.47–0.90)	1.31 (0.94–1.83)
Energy limits: 5th–95th centiles	17,973	443	0.69 (0.50–0.95)	0.64 (0.47–0.89)	1.35 (0.96–1.90)
Excluding deaths in two first years	17,919	431	0.69 (0.51–0.95)	0.66 (0.47–0.92)	1.26 (0.89–1.79)
Excluding special diet	16,620	406	0.73 (0.53–1.00)	0.65 (0.46–0.92)	1.30 (0.91–1.86)
Additionally adjusted for margarine	17,989	460	0.68 (0.51–0.93)	0.65 (0.47–0.90)	1.31 (0.94–1.83)
Additionally adjusted for other vegetable fats and oils	17,989	460	0.69 (0.51–0.93)	0.66 (0.47–0.90)	1.30 (0.93–1.83)
Additionally adjusted for prevalent depression	17,989	460	0.69 (0.53–0.98)	0.66 (0.48–0.91)	1.30 (0.93–1.82)
Including only cancer deaths	17,756	227	0.70 (0.45–1.08)	0.66 (0.42–1.05)	1.13 (0.69–1.83)
Including only CVD deaths	17,615	86	0.55 (0.28–1.18)	1.12 (0.42–2.96)	1.30 (0.64–2.66)
Without excluding participants with chronic diseases *	19,069	621	0.77 (0.59–1.00)	0.72 (0.54–0.95)	1.31 (0.98–1.76)

Adjusted for age, sex, BMI (kg/m^2^, linear and quadratic terms, continuous), marital status (dichotomous), years of university education (three categories), physical activity (continuous), total energy intake (five categories), cumulative smoking habit (packs–years, four categories), smoking status (three categories), alcohol intake (g/d), following a special diet (dichotomous), snacking (dichotomous), prevalent hypertension (dichotomous), and prevalent cholesterol (dichotomous). * Cardiovascular disease, diabetes, or cancer. Abbreviations: PVG: provegetarian; hPVG: healthful provegetarian; uPVG: unhealthful provegetarian; CVD: cardiovascular diseases.

## Data Availability

The information from the SUN Project that backs up our findings can be requested from the Department of Preventive Medicine and Public Health, School of Medicine, University of Navarra (Spain) at sun@unav.es.

## Supplementary Materials

The following supporting information can be downloaded at https://www.mdpi.com/article/10.3390/nu17152472/s1, Figure S1: Flow-chart of the study participants in the SUN cohort study; Table S1: Food groups included in the provegetarian (PVG) food pattern and scoring criteria for each food pattern (PVG, healthy PVG, and unhealthy PVG); Table S2: Macronutrient intake and food consumption of participants according to quintiles (Q) of the provegetarian (PVG), healthful provegetarian (hPVG), and unhealthful provegetarian (uPVG) food patterns in the SUN Project. Table S3: Pearson correlation coefficients between each provegetarian (PVG) food pattern and Mediterranean diet.

## Abbreviations

The following abbreviations are used in this manuscript:

PVG	Provegetarian
hPVG	Healthful provegetarian
uPVG	Unhealthful provegetarian
SUN	Seguimiento Universidad de Navarra
