# Supplementary material for: Adherence to the Provegetarian Food Patterns and Incidence of All-Cause Mortality in a Mediterranean Population: The SUN Cohort
Source: Nutrients. 2025 Jul 29;17(15):2472. doi: 10.3390/nu17152472 (PMC12348523; doi:10.3390/nu17152472)
Supplement: Supplementary file 1 [file nutrients-17-02472-s001.zip › nutrients-3734807-supplementary.pdf]

Figure S1. Flow-chart of the study participants in the SUN cohort study.

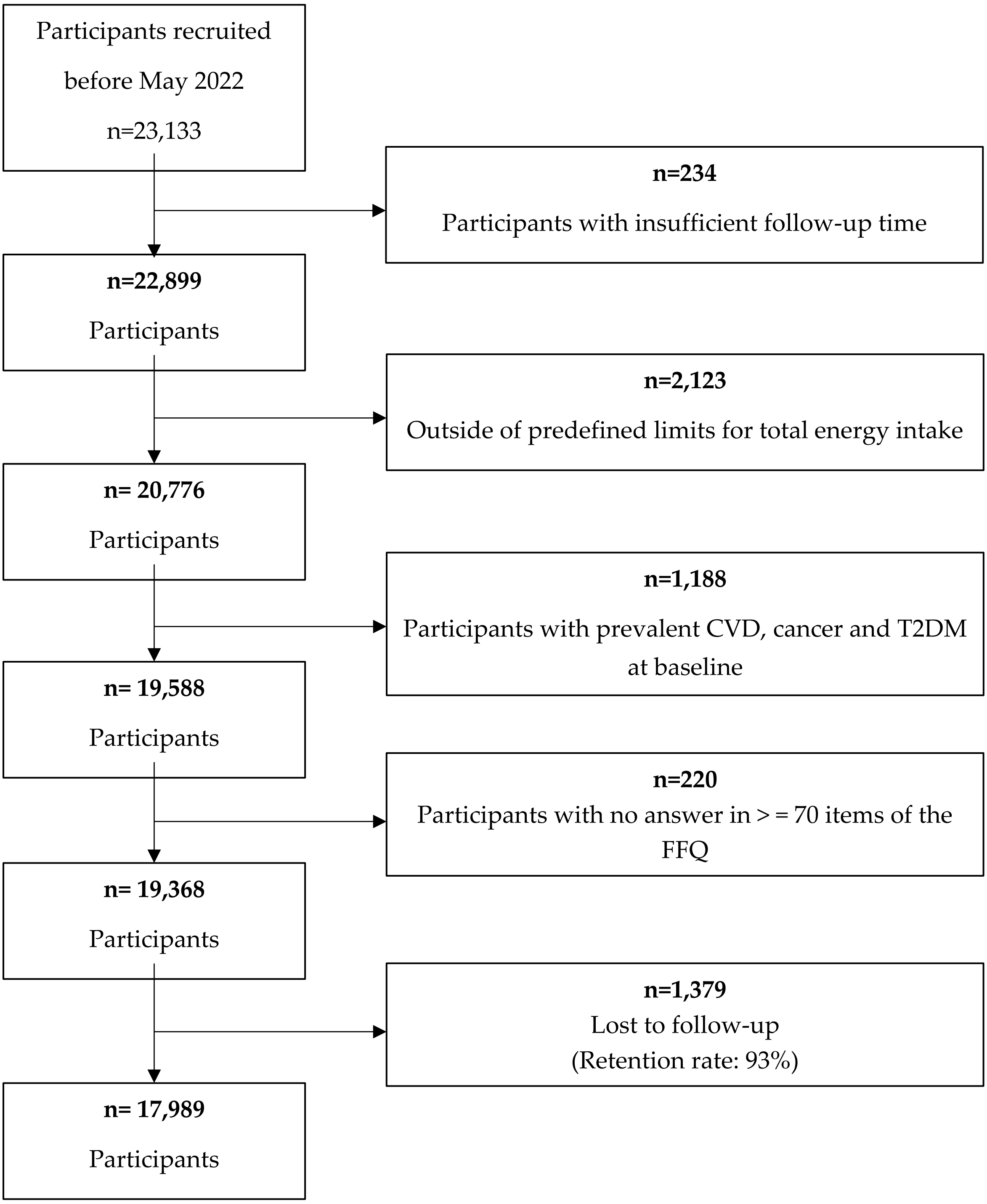

Abbreviations: T2DM: Type 2 diabetes.

Table S1. Food groups included in the provegetarian (PVG) food pattern and scoring criteria for each food pattern (PVG, healthy PVG and unhealthy PVG).

**Plant Food Groups**	**Foods included in each food group**	**PVG**	**hPVG**	**uPVG**
**Healthy**				
1.Vegetables	Swiss chard, spinach, cabbage, cauliflower, broccoli, lettuce, chicory, tomatoes, carrot, pumpkin, green beans, eggplant, zucchini, cucumber, pepper, asparagus, others	+	+	-
2.Fruits	Citrus, grapes, banana, apple, pear, strawberry, peach, apricot, nectarine, cherries, plums, figs, melon, watermelon, grapes, mango, papaya, kiwi, olives, avocado	+	+	-
3.Legumes	Lentils, chickpeas, beans, peas	+	+	-
4.Nuts	Almonds, peanuts, hazelnuts, walnuts	+	+	-
5.Olive oil	Olive oil	+	+	-
6.Whole grains	Whole-grain bread	+	+	-
7.Boiled or baked potatoes	Boiled or baked potatoes	+	+	-
8.Coffee	Coffee, decaffeinated coffee	+	+	-
**Unhealthy**				
9.Refined grains	White bread, cold breakfast cereal, rice, pasta	+	-	+
10.French fries	Potato chips, French fries	+	-	+
11.Sugar-sweetened beverages	Carbonated beverages with sugar, low calorie carbonated beverages	+	-	+
12.Fruit juices	Fresh orange juice, other natural fruit juices and bottled fruit or vegetable juices	+	-	+
13.Pastries	Cookies, home-baked and ready-made cakes, muffins, donuts, croissant, cakes, churros, chocolates, nougat, marzipan	+	-	+
**Animal Food Groups**		**PVG**	**hPVG**	**uPVG**

14.Dairy products	Skimmed milk, semi-skimmed milk, whole milk, condensed milk, cream, whole fat yogurt, low fat yogurt, ice cream, milkshakes, curd, cottage or white cheese, cheese in portions, custard, curd	-	-	-
15.Meat and meat products	Chicken, turkey beef, veal, pork, lamb, liver, other viscera, serrano ham, cooked ham, spicy pork sausage, salami, mortadella, foie gras, black pudding, hamburger, other cured or smoked meats, bacon, hot dog	-	-	-
16.Fish or seafood	White fish, blue fish, salted or smoked fish, clams, mussels, shrimp, squid, octopus	-	-	-
17.Eggs	Eggs	-	-	-
18.Animal fats	Butter, lard	-	-	-
19.Miscellaneous food	Instant soups, mayonnaise, croquettes, pasties	-	-	-

Abbreviations: PVG: Provegetarian hPVG: Healthful provegetarian; uPVG: unhealthful provegetarian; +: positive score; -: reverse score.

Table S2. Macronutrient intake and food consumption of participants according to quintiles (Q) of the Provegetarian (PVG), healthful provegetarian (hPVG), and unhealthful provegetarian (uPVG) food patterns in the SUN Project.

	PVG			hPVG			uPVG		
**Variables**	**Q1**	**Q2-Q4**	**Q5**	**Q1**	**Q2-Q4**	**Q5**	**Q1**	**Q2-Q4**	**Q5**
**N**	4278	10215	3496	4090	10634	3265	4130	10559	3300
Provegetarian score range	29-52	53-62	63-81	34-51	52-63	64-86	31-51	52-63	64-87
**Macronutrients (% E)**									
Carbohydrate intake (%E)	40.5 (7.1)	43.5 (7.0)	47.2 (6.8)	42.6 (6.5)	43.3 (7.3)	45.3 (8.0)	41.0 (7.3)	43.5 (7.1)	46.7 (7.0)
Protein intake (%E)	19.0 (3.3)	18.4 (3.1)	17.0 (2.9)	18.3 (3.0)	18.4 (3.3)	17.7 (3.1)	20.3 (3.1)	18.2 (2.9)	15.8 (2.4)
Fat intake (%E)	38.7 (6.4)	36.1 (6.3)	33.7 (6.3)	37.5 (5.6)	36.2 (6.5)	34.8 (7.2)	36.6 (6.5)	36.3 (6.5)	35.7 (6.5)
SFA (%E)	14.4 (3.2)	12.4 (2.8)	10.5 (2.7)	14.0 (2.9)	12.5 (3.0)	10.6 (3.0)	12.1 (3.1)	12.6 (3.2)	12.6 (3.1)
MUFA (%E)	16.3 (3.5)	15.7 (3.6)	15.2 (3.8)	15.6 (2.9)	15.8 (3.7)	16.0 (4.2)	16.4 (3.7)	15.8 (3.6)	15.0 (3.5)
PUFA (%E)	5.3 (1.7)	5.2 (1.5)	5.1 (1.5)	5.5 (1.5)	5.2 (1.5)	5.0 (1.5)	5.0 (1.3)	5.2 (1.5)	5.6 (1.8)
Trans fatty acid (%E)	0.4 (0.2)	0.4 (0.2)	0.3 (0.2)	0.4 (0.2)	0.4 (0.2)	0.3 (0.2)	0.3 (0.2)	0.4 (0.2)	0.4 (0.2)
Total dietary fiber (g/d)	20.3 (8.7)	22.5 (9.6)	25.6 (10.7)	17.0 (6.1)	21.7 (8.0)	32.1 (11.7)	25.1 (10.2)	21.9 (9.6)	21.4 (9.0)
**Food consumption**									
**Plant- based food groups**									
Vegetables (g/d)	459 (312)	529 (338)	615 (341)	362 (217)	519 (291)	770 (443)	669 (344)	518 (330)	390 (277)
Fruits (g/d)	342 (303)	410 (315)	488 (323)	263 (185)	395 (278)	638 (426)	479 (298)	401 (318)	347 324)
Legumes (g/d)	21 (19)	22 (17)	24 (16)	18 (12)	22 (17)	28 (22)	26 (17)	22 (17)	20 (17)
Nuts (g/d)	5 (8)	7 (12)	10 (14)	4 (5)	6 (9)	14 (19)	9 (13)	7 (11)	5 (10)

Olive oil (g/d)	17 (15)	18 (14)	20 (14)	13 (10)	18 (14)	25 (16)	20 (14)	18 (14)	17 (15)
Whole grains (g/d)	8 (28)	13 (30)	18 (33)	4 (14)	11 (26)	31 (47)	21 (38)	11 (29)	6 (23)
Refined grains (g/d)	95 (77)	88 (67)	86 (59)	97 (66)	88 (69)	82 (68)	59 (44)	87 (62)	133 (87)
Boiled or baked potatoes (g/d)	22 (25)	27 (29)	34 (31)	21 (23)	27 (29)	37 (34)	32 (30)	27 (29)	23 (27)
French fries (g/d)	29 (33)	25 (28)	23 (26)	36 (32)	24 (28)	16 (23)	13 17)	25 (27)	44 (38)
Coffee (servings/d)	1.3 (1.3)	1.5 (1.3)	1.7 (1.3)	1.1 (1.2)	1.5 (1.3)	1.7 (1.4)	1.8 (1.4)	1.4 (1.3)	1.2 (1.2)
Sugar sweetened beverages (servings/d)	0.3 (0.7)	0.3 (0.6)	0.3 (0.6)	0.5 (0.7)	0.3 (0.6)	0.2 (0.5)	0.2 (0.5)	0.3 (0.6)	0.5 (0.9)
Fruit juices (servings/d)	0.4 (0.6)	0.4 (0.6)	0.6 (0.7)	0.5 (0.6)	0.4 (0.6)	0.4 (0.7)	0.3 0.4	0.5 0.6	0.6 0.8
Pastries (g/d)	58 (50)	49 (43)	44 (37)	62 (43)	49 (44)	38 (38)	28 (24)	48 (37)	84 (60)
**Animal food groups**									
Dairy products (g/d)	551 (294)	411 (235)	297 (198)	464 (247)	416 (257)	389 (267)	435 (254)	419 (262)	415 (251)
Meat and meat products (g/d)	226 (83)	172 (68)	126 (62)	202 (76)	174 (76)	151 (77)	176 (75)	175 (78)	180 (78)
Fish or seafood (g/d)	108 (67)	97 (57)	85 (55)	92 (53)	97 (58)	107 (70)	121 (61)	95 (59)	76 (48)
Eggs (g/d)	29 (20)	22 (13)	16 (11)	28 (18)	22 (15)	19 (13)	25 (14)	23 (16)	22 (16)
Animal fats (g/d)	1.8 (3.4)	1.0 (2.5)	0.5 (1.6)	1.7 (3.2)	1.0 (2.5)	0.5 (2.1)	1.1 (2.6)	1.1 (2.6)	1.1 (2.8)
Miscellaneous food* (g/d)	44 (56)	28 (37)	18 (29)	44 (47)	28 (42)	17 (29)	27 (35)	30 (43)	31 (46)

Abbreviations: PVG: Provegetarian; hPVG: Healthful provegetarian; uPVG: unhealthful provegetarian; SFA: Saturated Fatty Acids; MUFA: Monounsaturated Fatty Acids; PUFA: Polyunsaturated Fatty Acids; %E: % of total energy intake.

*Miscellaneous foods: croquettes, pasties, instant soups and mayonnaise.

Table S3. Pearson correlation coefficients between each provegetarian (PVG) food pattern and Mediterranean diet.

	MedDiet	PVG	hPVG	uPVG
**MedDiet**	1.00			
**PVG**	0.38	1.00		
**hPVG**	0.54	0.57	1.00	
**uPVG**	-0.29	-0.06	-0.49	1.00

P value <0.05 for all correlation coefficients

Abbreviations: PVG: Provegetarian; hPVG: Healthful provegetarian; uPVG: unhealthful provegetarian.
